# Native Desorption Electrospray Ionization Liberates Soluble and Membrane Protein Complexes from Surfaces

**DOI:** 10.1002/anie.201704849

**Published:** 2017-09-18

**Authors:** Stephen Ambrose, Nicholas G. Housden, Kallol Gupta, Jieyuan Fan, Paul White, Hsin‐Yung Yen, Julien Marcoux, Colin Kleanthous, Jonathan T. S. Hopper, Carol V. Robinson

**Affiliations:** ^1^ Department of Chemistry, Physical & Theoretical Chemistry Laboratory University of Oxford Oxford OX1 3QZ UK; ^2^ Department of Biochemistry University of Oxford Oxford UK; ^3^ Current address: IPBS CNRS, UMR 5089 205 Route de Narbonne 31077 Toulouse France

**Keywords:** desorption, electrospray ionisation, G-protein coupled receptors, mass spectrometry, membrane protein complexes

## Abstract

Mass spectrometry (MS) applications for intact protein complexes typically require electrospray (ES) ionization and have not been achieved via direct desorption from surfaces. Desorption ES ionization (DESI) MS has however transformed the study of tissue surfaces through release and characterisation of small molecules. Motivated by the desire to screen for ligand binding to intact protein complexes we report the development of a native DESI platform. By establishing conditions that preserve non‐covalent interactions we exploit the surface to capture a rapid turnover enzyme–substrate complex and to optimise detergents for membrane protein study. We demonstrate binding of lipids and drugs to membrane proteins deposited on surfaces and selectivity from a mix of related agonists for specific binding to a GPCR. Overall therefore we introduce this native DESI platform with the potential for high‐throughput ligand screening of some of the most challenging drug targets including GPCRs.

A range of applications, including 2D imaging of small molecules and metabolites released from tissue cross‐sections, has become possible with the introduction of powerful DESI approaches when coupled with MS.^1^, ^2^ The primary goal of DESI applications has been to focus on the small molecules released, for example in the real‐time detection of tumour tissue during surgical procedures.^3^, ^4^ While DESI has also been adapted to study large biomolecules, via the mixing of ES droplets and solution in a process known as liquid DESI,^5^, ^6^ it has not yet been applied to proteins deposited on surfaces and desorbed in solutions that retain their native state interactions. Despite considerable progress in applications of non‐denaturing or native MS (nMS) of soluble^7^, ^8^, ^9^ and membrane embedded proteins^10^ the possibility of effectively “lifting” intact complexes from surfaces is desirable since many high throughput technologies then become accessible.^11^ Moreover the lipid distribution in natural membranes is essentially planar and asymmetric with varying spatial and temporal arrangements in the vicinity of embedded protein complexes.^12^ The lipid distribution and the desire for a surface technology that is also able to analyse membrane proteins motivated us to develop a modified DESI approach capable of releasing folded protein molecules from planar surfaces and to construct an interface that we could couple to a high‐resolution Orbitrap MS optimised for high mass transmission^13^ of membrane proteins.^14^ We demonstrate the potential of this methodology in three ways 1) by capturing transient protein substrate products 2) by screening for optimal solution/purification conditions using picoMoles of membrane and soluble proteins and 3) by carrying out ligand binding experiments on planar surfaces.

We modified the design of the original DESI set‐up^1^ and with our custom‐built ion source, ES device and sample stage coupled our interface to an Orbitrap Q‐Exactive (Figure 1 a and Supporting Information Figure S1). We found that signal intensity was significantly improved if the length of the sample transfer tube, used in conventional DESI set‐ups,^15^ was minimised and the stage was located directly under the inlet of our ion source. We optimised signal intensity using hen egg‐white lysozyme and found that the spectra recorded under these native DESI conditions are similar to those from typical nanoflow ES capillaries, implying that the folded state of the protein is maintained (Figure 1 b). If the native fold is maintained then deposited lysozyme should be able to carry out enzymatic functions. Accordingly with *N*‐acetyl‐glucosamine (NAG) substrate added to the desorption spray and directed at lysozyme deposited on the stage we observed additional peaks assigned to binding of intact NAG‐5 to lysozyme (Figure 1 c). The rapid turnover of this substrate precludes its observation in solution‐based ES^16^ but since substrate binding takes place during rapid desorption, analogous to reactive DESI experiments reported for small molecules,^17^ the transient bound state can be captured using this native DESI approach.

**Figure 1:**
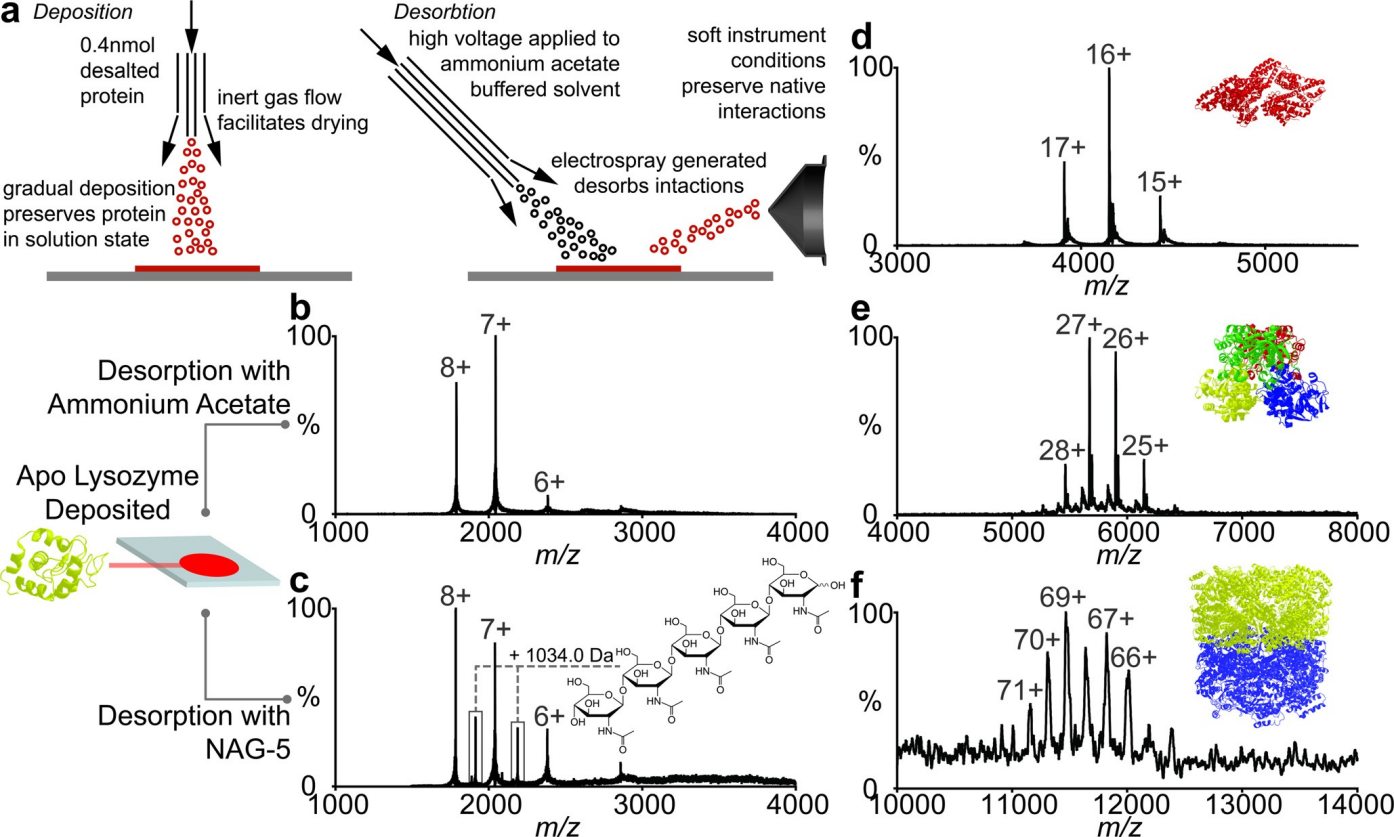
Schematic of the native DESI setup showing deposition of protein on the stage, followed by desorption and analysis in the mass spectrometer with representative spectra for a series of soluble protein their substrates and complexes. a) First, protein is deposited on the native DESI stage (red) from aqueous buffer and second, the ES plume is charged with a voltage of 2.5–3.5 V and directed at the stage. Transfer is effected by positioning the stage close to the orifice of the mass spectrometer. b) Apo lysozyme is deposited on the stage (25 μL, 10 μm) in aqueous ammonium acetate (200 mm, pH 6.8) and the same buffer is used to desorb the protein. c) NAG‐5 is added to the ES plume directed at the lysozyme deposit, additional peaks reveal binding of the substrate NAG‐5 prior to its cleavage. Native DESI mass spectra of d) monomeric bovine serum albumin, e) tetrameric alcohol dehydrogenase and f) the GroEL_14‐mer._

To investigate application to larger protein assemblies we chose complexes with a range of oligomeric states: bovine serum albumin, tetrameric alcohol dehydrogenase (ADH) and the GroEL_14‐mer_. We were able to record native DESI mass spectra for all three with masses of 66, 148 and 800 kDa, respectively and with established subunit stoichiometries (Figure 1 d,e,f). These results effectively transform DESI from a small molecule approach to a method capable of detecting intact protein assemblies from surfaces. In this regard an obvious next target is membrane proteins since their natural environment is in lipid bilayers.

For this study we selected the outer‐membrane protein F (OmpF), a trimer of transmembrane beta‐barrels. We deposited 0.4 nmol of OmpF in ammonium acetate (200 mm) containing octyl glucoside (OG) micelles and directed the desorption plume at the deposited protein. Initially a relatively low intensity signal was observed (Figure 2 a) followed by rapid deterioration and loss of signal—indicating a dilution effect at the surface leading to disruption of the micelle.^18^ Adding OG to the desorption solution (at twice the critical micelle concentration (cmc) (1 % w/v OG) we observed recovery of the OmpF trimer signal (Figure 2 b) which remained stable for 30 mins (Figure S2).^18^ Substituting a different detergent (Lauryldimethylamine *N*‐oxide (LDAO) 0.05 % w/v) into the desorption plume induces a shift to higher charge states (Figure 2 c), observed previously with LDAO^19^ implying that detergent exchange has occurred on the stage. This highlights an important capability of the native DESI approach for rapid detergent screening.

**Figure 2:**
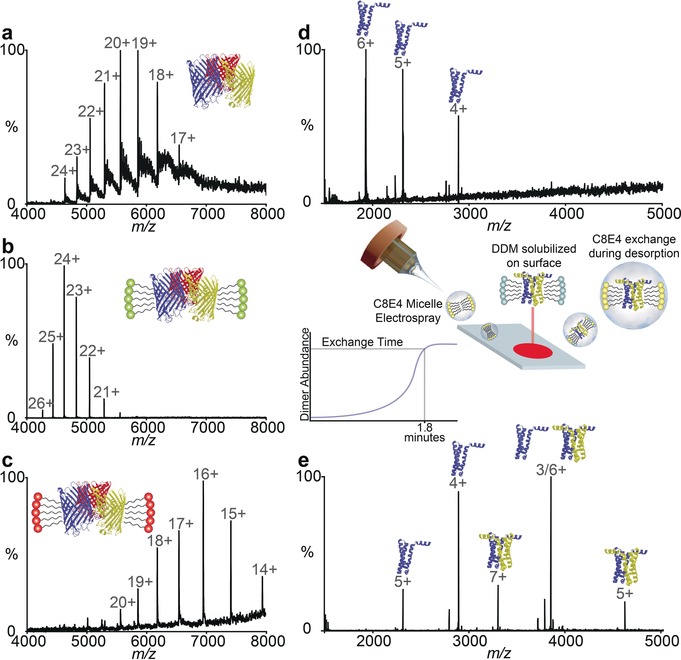
Native DESI of membrane proteins reveals sensitivity to their detergent environment. a) OmpF deposited in OG but without detergent in the ES plume leads to rapid deterioration of signal and an average charge state of ≈20+. b) Adding OG to the ES plume recovers mass spectra of OmpF trimer with an average charge state of ≈24+. c) Detergent exchange into LDAO on the DESI target reduces the average charge state of the OmpF trimer to ≈16+. d) SemiSWEET desorbed with a DDM containing buffer yields a mass spectrum of monomeric protein (6+) while addition of C8E4 to the desorption buffer yields a population of dimeric protein.

Screening for optimal detergents, as part of membrane protein purification protocols, is time consuming and uses valuable protein resources.^20^ We investigated further the possibility of detergent screening on the DESI stage using the sugar transporter semiSWEET from *Vibrio splendidus* 21 since it is extremely sensitive to its detergent environment. Previous MS experiments established that this transporter exists in an entirely monomeric form in DDM and exhibits a monomer–dimer equilibrium in the detergent (C8E4).^22^ Depositing semiSWEET on the DESI stage in DDM (Figure 2 d) we then added C8E4 (0.5 % w/v) directly to the desorption buffer. After 1.8 min of desorption with the C8E4‐containing buffer the total ion chromatogram for the (7+) ion (indicative of the dimer) was observed consistent with detergent exchange on the DESI stage from the initial DDM conditions to the C8E4 micelles (Figure 2 e). Detergent screening on the DESI stage, with minimal membrane protein consumption, highlights a powerful feature of this approach.

Turing to ligand screening an important criterion to establish is the extent to which protein complexes dissociate into their components during native DESI as opposed to conventional nanoflow ES. Selecting an outer membrane protein receptor FpvA from *Pseudomonas aeruginosa,* which translocates ferric‐pyoverdine (Pvd) across outer membranes,^23^ we formed the complex in solution and compared the percentage of complex FpvA:PvD using the different ionization methods. We found 59 % and 61 % in the native DESI and nanoES approaches, respectively (Figure 3 a and b). We conclude that our native DESI approach, which involves both deposition and desorption within protective micelles, reproduces the nano‐ES result in which complexes are electrosprayed directly from solution.

**Figure 3:**
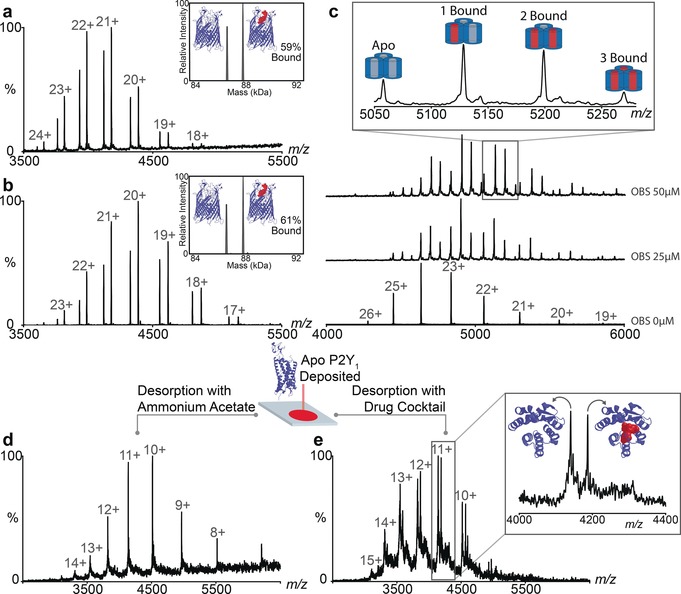
The extent of complex formation is comparable to that observed in nano‐ES and enables determination of *K*_d_ values and competitive binding experiments. Comparison of mass spectra for the complex FvpA:FvD recorded by a) DESI and b) nESI. c) Titration of the peptide OBS1 to OmpF at 0, 25 μm and 50 μm for determination of *K*_d_. d) Deposition of the GPCR P2Y_1_ and desorption in a mixed micelle and e) competitive binding of the specific ligand from a cocktail of six ligands.

Exploring further the quantitative aspects of this native DESI platform we selected OBS1 a 17‐residue peptide known to bind within the pores of OmpF with binding constants determined previously by both ITC and nanoES MS.^24^, ^25^ We deposited OmpF on the stage in OG detergent and incubated OmpF with increasing concentrations of OBS1 (0–75 μm) and deposited these protein‐peptide complexes onto the native DESI stage. The relative intensities of bound to unbound protein were extracted and plotted as a function of OBS1 concentration (Figure S3). The *K*_d_ determined (0.7±0.34 μm) for this membrane protein complex is in agreement with values reported (1.0±0.1 μm).^24^ That the solution state equilibria is maintained is surprising given that DESI involves ejection of the membrane protein complex, deposited on the surface, by a desorption spray. However detailed kinetics of OmpF–OBS1 peptide complexes are not known so it is unclear how this reflects the kinetics of DESI.

Beyond peptide binding established in solution we wanted to perform binding experiments on membrane proteins deposited on the stage. Delipidated *apo* OmpF (20 μm) was deposted and OBS1 added to the desorption buffer. Up to three peptides bound per trimer were detected with the predominant peak corresponding to two (Figure S3 a). This experiment confirms that the peptide can access binding sites within the short time frame during desorption, even in the presence of detergent. Similarly we added phosphatidylglycerol lipid (POPG) to the desorption buffer, at a concentration of 5 μm, and observed up to three lipids bound to OmpF (Figure S3 b). OmpF is also reported to bind to a range of antibiotics within the extracellular and periplasmic pore vestibule.^26^ Addition of kanamycin (50 μm) to the desorption buffer reveals binding of one molecule of the antibiotic per OmpF trimer (Figure S3 c). Since we anticipate that the peptide binds within the pore, while the lipids bind to the outer surface and kanamycin to the top of the pore, we have highlighted our capability to bind directly to the membrane protein via three different mechanisms. In each of these three scenarios we assume that the small molecule has not reached solution phase equilibrium, since desorption is rapid, but rather has penetrated to some extent the protective micelle that surrounds the membrane protein while deposited on the surface.

Building on this ability to screen for binding to a membrane protein target deposited on a surface we reasoned that it would be possible to add multiple ligands simultaneously. To explore this possibility we selected a Class A G‐protein couple receptor (GPCRs) depositing onto the stage 0.4 nmoles of P2Y_1_, responsible for platelet aggregation and a key target for anti‐thrombotic therapy.^27^ After recording a native DESI spectrum in its *apo* form we added a cocktail of antagonists/agonists designed to target related GPCRs (Figure 3 d,e and Table S1). The native DESI spectrum reveals a discrete mass increase (560.03 Da) in exact agreement with the mass of MRS2500 (1′R,2′S,4′S,5′S)‐4‐(2‐Iodo‐6‐methyl amino‐purine‐9‐yl)‐1‐[(phosphato)methyl]‐2‐(phosphato)bicycle[3.1.0]‐hexane bound to P2Y_1_. No other adducts were observed and control experiments, where the specific inhibitor was excluded from the drug cocktail, revealed no ligand binding to P2Y_1_ (Figure S6). These results reveal that this native DESI platform is capable of detecting selective binding of a specific antagonist to a GPCR from a multicomponent mixture.

In summary we have developed and applied a native DESI platform and shown that it is capable of preserving the native structure of both soluble and membrane proteins and their complexes. Comparing our approach with ambient ionization methods described previously we note the addition of chemicals in the spray solution in reactive DESI applications for small molecule analyses.^17^, ^28^ Protein ligand binding experiments have also been achieved with reactions taking place by mixing in solution in a liquid sample DESI approach^6^ rather than by interaction following protein deposition on the planar target as shown here. Moreover kinetic approaches have been developed using mixing experiments to monitor the small molecules released during enzymatic cleavage by means of liquid DESI and have increased the range of buffers that can be used.^29^, ^30^ Our native DESI approach is largely restricted to volatile buffers and detergents that have been optimised for native MS. A further limitation is imposed by the fact that ligands are observed directly bound to proteins desorbed from a planar surface, high‐resolution MS is therefore critical.

The most exciting aspect of this native DESI approach however, is the potential of our method to study intact membrane proteins and their complexes. The ability to place membrane protein targets on planar surfaces in different lipidic environments, without tethering the proteins, and to carry out selective binding from a cocktail of drugs offers possibilities for high throughput screening. Many downstream applications become accessible including the ability to carry out multiple experiments on the same target; for example detergent optimisation, the screening of multiple lipids and ligands that bind to a drug target or the trapping of fast turnover products in enzyme catalysed reactions. Analogous to the powerful native MS methods, now widely accepted as a key component in structural biology, native DESI enables further possibilities for development of spatial, temporal and even directional analyses within artificial bilayers or membrane mimetics.

## Conflict of interest

The authors declare no conflict of interest.

## Supporting information

As a service to our authors and readers, this journal provides supporting information supplied by the authors. Such materials are peer reviewed and may be re‐organized for online delivery, but are not copy‐edited or typeset. Technical support issues arising from supporting information (other than missing files) should be addressed to the authors.

Supplementary
