# Supplementary material for: Native Desorption Electrospray Ionization Liberates Soluble and Membrane Protein Complexes from Surfaces
Source: Angew Chem Int Ed Engl. 2017 Sep 18;56(46):14463–8. doi: 10.1002/anie.201704849 (PMC5813186; doi:10.1002/anie.201704849)
Supplement: Supplementary file 1 — Supplementary [file ANIE-56-14463-s001.pdf]

Angewandte Chemie
Eine Zeitschrift der Gesellschaft Deutscher Chemiker

Supporting Information

Native Desorption Electrospray Ionization Liberates Soluble and Membrane Protein Complexes from Surfaces

Stephen Ambrose, Nicholas G. Housden, Kallol Gupta, Jieyuan Fan, Paul White, Hsin-Yung Yen, Julien Marcoux, Colin Kleanthous, Jonathan T. S. Hopper, and Carol V. Robinson**

anie_201704849_sm_miscellaneous_information.pdf

Supplementary Information

Materials and Methods

Non Denaturing MS

An in-house native DESI source was designed to interface with a Q Exactive mass spectrometer (Thermo Fisher Scientific) that had been modified for the transmission and detection of higher m/z ions with the following settings: a higher energy collisional dissociation (HCD) cell pressure was set between 0.7 and 1.2 x 10^{-9} mbar to allow the transmission of smaller and larger complexes respectively. An ion transfer capillary temperature of 60 °C promoted soft desolvation of ions. The cone voltage was typically set to 20V, unless additional activation was required with certain maltoside detergents in which case it was raised to 100V. The S-lens RF potential was maintained at 100V. The quadrupole was kept at a full scan range to allow transmission of ions up to ~20,000 m/z. The HCD energy was typically kept at 0V for soluble proteins and between 120 and 200V for membrane proteins with more (C8E4) or less (DDM) labile detergents. Nano-electrospray spectra were obtained under identical instrument conditions, but gold-coated capillaries were used in place of the DESI stage and source. No nebulizing gas flow was used and capillary voltages were typically 1.2-.1.5kV.

The native DESI sprayer source was constructed from a stainless steel tee union (Swagelock) containing a 75µm-i.d./360µm-o.d. fused-silica capillary (Upchurch Scientific) surrounded by a 500µm-i.d./1588µm-o.d. PEEK sheath (Upchurch Scientific). The spray solvent typically consisted of a 200mM ammonium acetate solution supplemented with ligands and/or detergent where stated, which was supplied by a 50 µL gas-tight syringe (Hamilton) at a flow rate of 2.5 µLmin^{-1}. A high voltage of 3-4 kV was applied to the solution through a liquid junction. The nitrogen source to the sheath was typically maintained at 65 psi. The sprayer was manipulated by x-y-z translation and rotation stages (Newport) with typical parameters of: incident angle = 60°, tip-to-surface distance = 3 mm, sample-to-inlet distance = 2 mm.

Samples were prepared for DESI analysis by gentle deposition with a nebulizing sprayer. Glass stages were prepared by UV-oxidation followed by rinsing in acetone then doubly distilled H_2O to ensure a clean surface. 25 µL of desalted protein was then deposited with a nebulizing sprayer identical to the one described above, with the following differences. No voltage was applied during sample deposition and the source was perpendicular to the surface at a distance of 10 mm. A sample flow rate of 1µL min^{-1} and nebulizing N_2 pressure of 35 psi was used to minimize sample dispersion on the surface. Using these conditions reproducibly gave even sample deposition within a ~3 mm diameter spot.

Protein Expression and Purification

E. coli Outer Membrane Porin (OmpF) was expressed in BE3000 cells and purified in octyl-glucoside (OG) as previously described [21]. *V. splendidus* SemiSWEET was expressed in BL21 (DE3) cells and purified in n-Dodecyl-β-D-Maltopyranoside (DDM) as previously described [18].

The $P2Y_1$ receptor was overexpressed in insect cells and the cell membranes were enriched as previously described [24].

FpvA was expressed in *E. coli* TNE012 cells and was purified from outer membranes using a modified BtuB purification protocol [27]. FpvA was purified using DEAE-Sepharose anion exchange chromatography, Sephacryl S300 gel filtration and MonoQ anion exchange chromatography. The Ga^{3+}-bound form of Pvd, purchased from Sigma Aldrich, was added to purified FpvA and the complex was purified to homogeneity on a Superdex 200 10/300 GL column (GE Healthcare) in 200 mM ammonium acetate, 1% w/v OG.

Prior to MS analysis, protein samples were desalted and buffer-exchanged into 200 mM ammonium acetate (unless otherwise stated) using biospin-6 (Bio-Rad) and diluted to 15 µM.

Detergent Exchange

Detergent exchange was achieved using our native DESI platform by depositing semiSWEET solubilized in 0.02% (w/v) DDM detergent onto the DESI stage. Protein was then desorbed using 200 mM ammonium acetate with 0.5% (w/v) C8E4 and the peak intensities monitored. The abundance of the dimer as a function of DESI time was determined by extracting the relative intensity of the peak at m/z 3303 – the predominant dimer peak that doesn't overlap with monomer peaks.

Binding Measurements

FvpA in complex with pyoverdine was desorbed from a solution of 200 mM ammonium acetate and 1% (w/v) OG detergent. The same protein complex in the same solution conditions was subsequently

measured by nESI. For both experiments the spectra were deconvoluted using the UniDec software tool [28] to give a percentage of holo species relative to apo + holo.
OmpF was deposited at 15 µM with a range of OBS1 peptide (NH_2-^{2}SGGDGRGHNTGAHSTSG^{18}-$CONH_2$) (Severn Biotech) concentrations (0-75 µM). Protein peptide complexes were desorbed using 25 mM ammonium acetate with 1% (w/v) OG detergent. The spectra recorded at each concentration were deconvoluted using UniDec [28] and the mole fractions of the *apo*, OmpF 1, 2 and 3 peptide-bound peaks were collected from each measurement. A K_D was fitted to the data to give a single fixed value for the successive binding events.

For OmpF screening experiments apo protein was deposited onto the stage and desorbed with a 200 mM ammonium acetate buffered solution supplemented with 1% (w/v) OG detergent and the appropriate ligand. The lipids 1-palmitoyl-2-oleoyl-sn-glycero-3-phospho-(1'-rac-glycerol) (POPG) and 1-palmitoyl-2-oleoyl-sn-glycero-3-phosphocholine (POPC) were prepared from powder (Avanti Polar Lipids) at 10mg/ml as previously described and diluted 1:50 in the desorption solution. Kanamycin A sulfate (Sigma-Aldrich) was prepared as a 1mM stock in dd-H2O and added to the desorption solution at 50 µM. OBS1 peptide was prepared as a 1mM stock in dd-H2O and diluted to 25 µM in the desorption solution.
$P2Y_1$ drug screening experiments were performed by depositing apo protein and desorbing with a mixed micelle containing DDM, foscholine and cholesterol. The antagonists listed in s**upplementary table 1**) were selected to represent a candidate pool but only one component is the active antagonist (TOCRIS Bio-Techne). All ligands were prepared as 1mM stock solutions in dd-H_2O. The GPCR was then desorbed with a solution containing all antagonists at 20µM. A control experiment was performed by desorbing with all antagonists except MRS2500.

Supplementary Table 1

Drug	Structure	Mass	Target
MRS2500	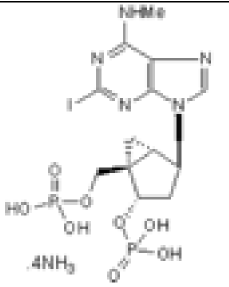	561.16	$P2Y_1$
MRS2211	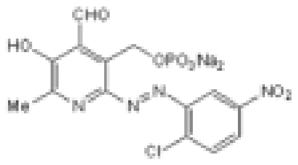	428.68	$P2Y_{13}$
PSB 0739	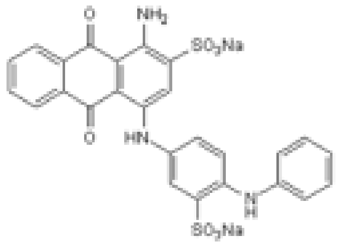	563.56	$P2Y_{12}$
PSB 1115	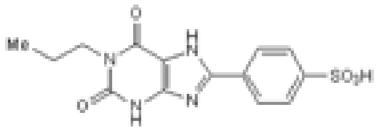	350.35	A2B
Ticlopidine Hydrochloride	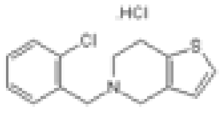	263.79	$P2Y_{12}$

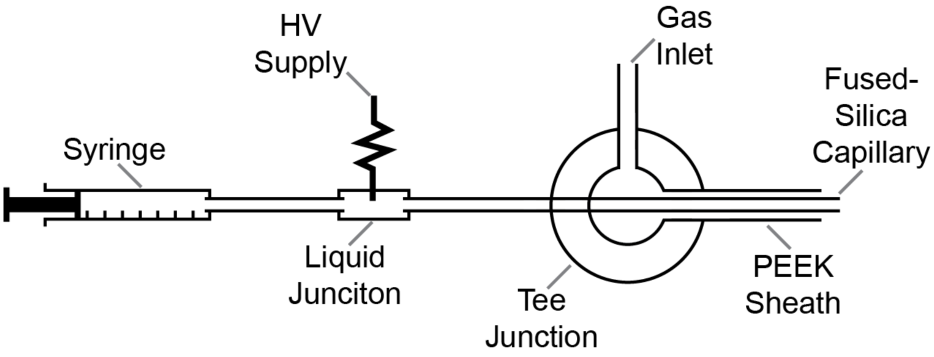

Figure S1 Schematic of the DESI sprayer comprising a gastight syringe containing the protein solution or spray solution for deposition or desorption respectively. The liquid junction joins the two separate capillary sections - one from the syringe pump and the other to the sprayer - meeting in a small volume tee-junction that has a gold wire inserted, to enable the voltage to be applied to the solution itself and not the surrounding fused silica capillary which is nonconductive. The tee-junction allows application of a nebulizing nitrogen gas flow into a PEEK sheath that envelops the capillary. The orientation of the sprayer is adjustable relative to the deposition stage, typically maintained at 90° or 60° during deposition or desorption respectively.

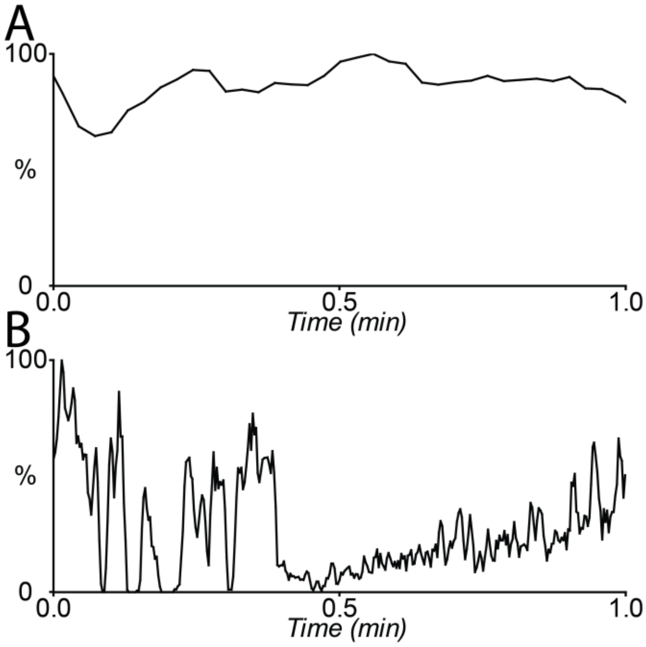

Figure S2 Total ion chromatograms for peaks at m/z 5298 which corresponds to the 21+ peak of the intact OmpF trimer desorbed using a solution containing either octylglucoside (1%) (A) or ammonium acetate (200mM) (B). Absence of a stable signal for desorption with ammonium acetate confirms the requirement for the detergent micelle for desorption of the membrane protein trimer OmpF.

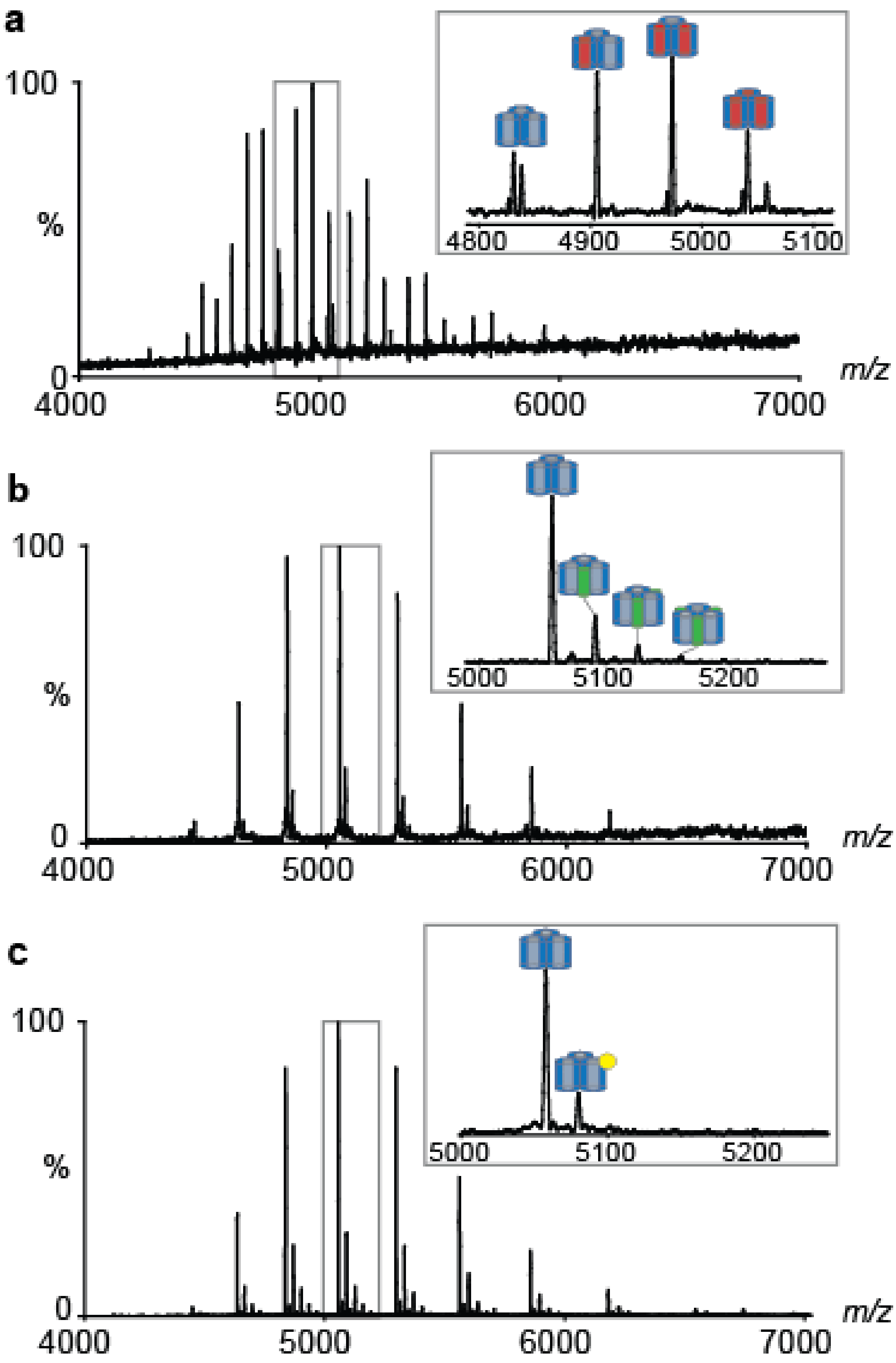

Figure S3 OmpF desorbed with small molecules that that thread through the centre, bind to the outside and at the top of the β–barrel protein channels. OmpF was deposited onto the DESI stage at a concentration of (15μM) and desorbed with OG (1%) solution containing the specific binding peptide OBS1 [29] in solution at a concentration of (50μM) (a) with the lipid POPG (5μM) (b) and with the antibiotic kanamycin (50μM) (c).

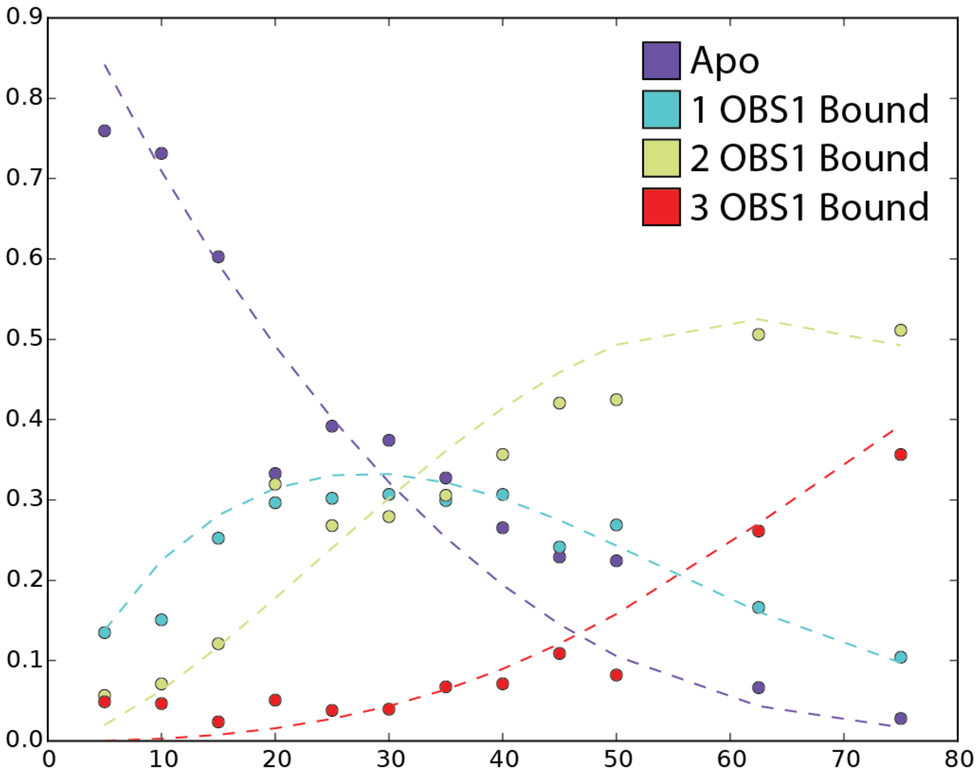

Figure S4 Determination of the K_d for OmpF binding to OBS1. The relative summed intensities of each species are plotted against the concentration of OBS. Kd fits generated with UniDec are also plotted, and a Kd value of 0.70 ± 0.34 µM is calculated and compares with values obtained previously via mass spectrometry [30,31] or ITC measurements [29].

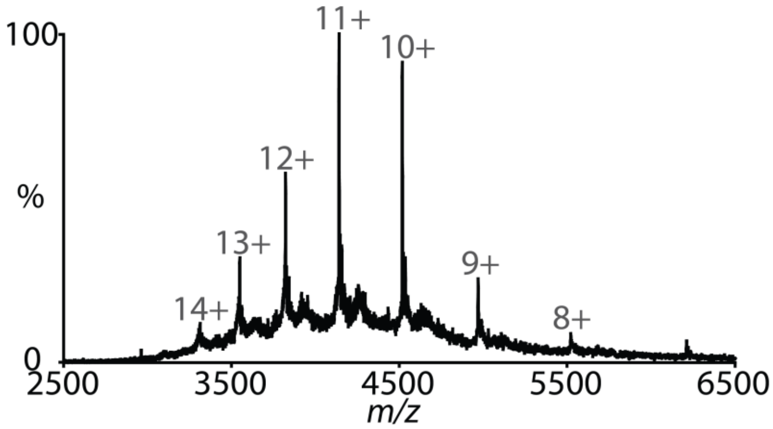

Figure S5 native DESI mass spectrum of the class A GPCR P2Y$_1$ liberated from the DESI stage using a mixed micelle containing DDM, foscholine and cholesterol. In the absence of MRS2500 no drug binding is observed confirming the selectivity of the receptor for its cognate ligand and highlighting the absence of non-specific binding of related ligands.

1. Housden, N. G., Wojdyla, J. A., Korczynska, J., Grishkovskaya, I., Kirkpatrick, N., Brzozowski, A. M. & Kleanthous, C. (2010) Directed epitope delivery across the Escherichia coli outer membrane through the porin OmpF, *Proc Natl Acad Sci U S A.* **107**, 21412-7.
2. Housden, N. G., Hopper, J. T. S., Lukoyanova, N., Rodriguez-Larrea, D., Wojdyla, J. A., Klein, A., Kaminska, R., Bayley, H., Saibil, H. R., Robinson, C. V. & Kleanthous, C. (2013) Intrinsically Disordered Protein Threads Through the Bacterial Outer-Membrane Porin OmpF, *Science.* **340**, 1570-1574.
3. Gault, J., Donlan, J. A. C., Liko, I., Hopper, J. T. S., Gupta, K., Housden, N. G., Struwe, W. B., Marty, M. T., Mize, T., Bechara, C., Zhu, Y., Wu, B., Kleanthous, C., Belov, M., Damoc, E., Makarov, A. & Robinson, C. V. (2016) High-resolution mass spectrometry of small molecules bound to membrane proteins, *Nature methods.* **13**, 333-336.
